# Cumin anaphylaxis and allergy to spices in pediatrics: a case report and literature review

**DOI:** 10.3389/fimmu.2026.1838155

**Published:** 2026-07-06

**Authors:** Benedetta Pessina, Enrico Masiello, Mattia Giovannini, Maria Chiara Bardasi, Simona Barni, Francesco Catamerò, Angela Klain, Giulia Liccioli, Michele Miraglia Del Giudice, Elio Novembre, Lucrezia Sarti, Leonardo Tomei, Claudia Valleriani, Francesca Mori

**Affiliations:** 1 Pediatric Unit, Rho and Garbagnate Milanese Hospital, Azienda Socio Sanitaria Territoriale (ASST)-Rhodense, Milan, Italy; 2 Pediatric Unit Dipartimento di Emergenza e Accettazione (DEA) I “Presidio Ospedaliero (P.O.) Dono Svizzero” Formia, Asl Latina Sud, Latina, Italy; 3 Department of Health Sciences, University of Florence, Florence, Italy; 4 Allergy Unit, Meyer Children’s Hospital IRCCS, Florence, Italy; 5 Pediatric Unit, Università degli Studi di Ferrara, Ferrara, Italy; 6 Dipartimento della Donna, del Bambino e di Chirurgia Generale e Specialistica, Università degli Studi della Campania “Luigi Vanvitelli”, Naples, Italy; 7 Immunology Laboratory, Meyer Children’s Hospital IRCCS, Florence, Italy

**Keywords:** anaphylaxis, cumin, food allergy, food-induced anaphylaxis, inhalation, pediatric, spice allergy, spices

## Abstract

While spice allergies are rarely documented in children, spices are becoming an increasingly common allergen in pre-packaged foods. Current literature reports contradictory findings regarding the risk of severe reactions in pediatric patients, with some evidence suggesting that small amounts of spices usually used in home-cooked meals may be safe for children. However, allergens, such as spices, are increasingly used in the food industry, coupled with diverse culinary habits and customs, necessitates a re-evaluation of this risk. To the best of our knowledge, this is the first report of cumin anaphylaxis via inhalation in a pediatric patient. Spice allergies should be suspected in patients with allergic manifestations occurring briefly after the consumption or inhalation of multiple different and unrelated foods. Our experience indicates that prick-by-prick tests with spice powders are helpful and should be recommended as part of the diagnostic workup to investigate this peculiar type of food allergy and prevent misdiagnosis of idiopathic anaphylaxis. Limited data concerning the reliability of skin tests with native spices and extracts are available in the literature. Native spices could contain irritant agents leading to false-positive results, but standardized case-control studies are currently lacking. New techniques can be used in the diagnostic process; however, they still need to be validated and integrated into clinical practice. Further investigation is required to quantify the impact of spice allergies on the pediatric population, especially in today’s multiethnic societies.

## 1 Introduction

**Figure 1:**
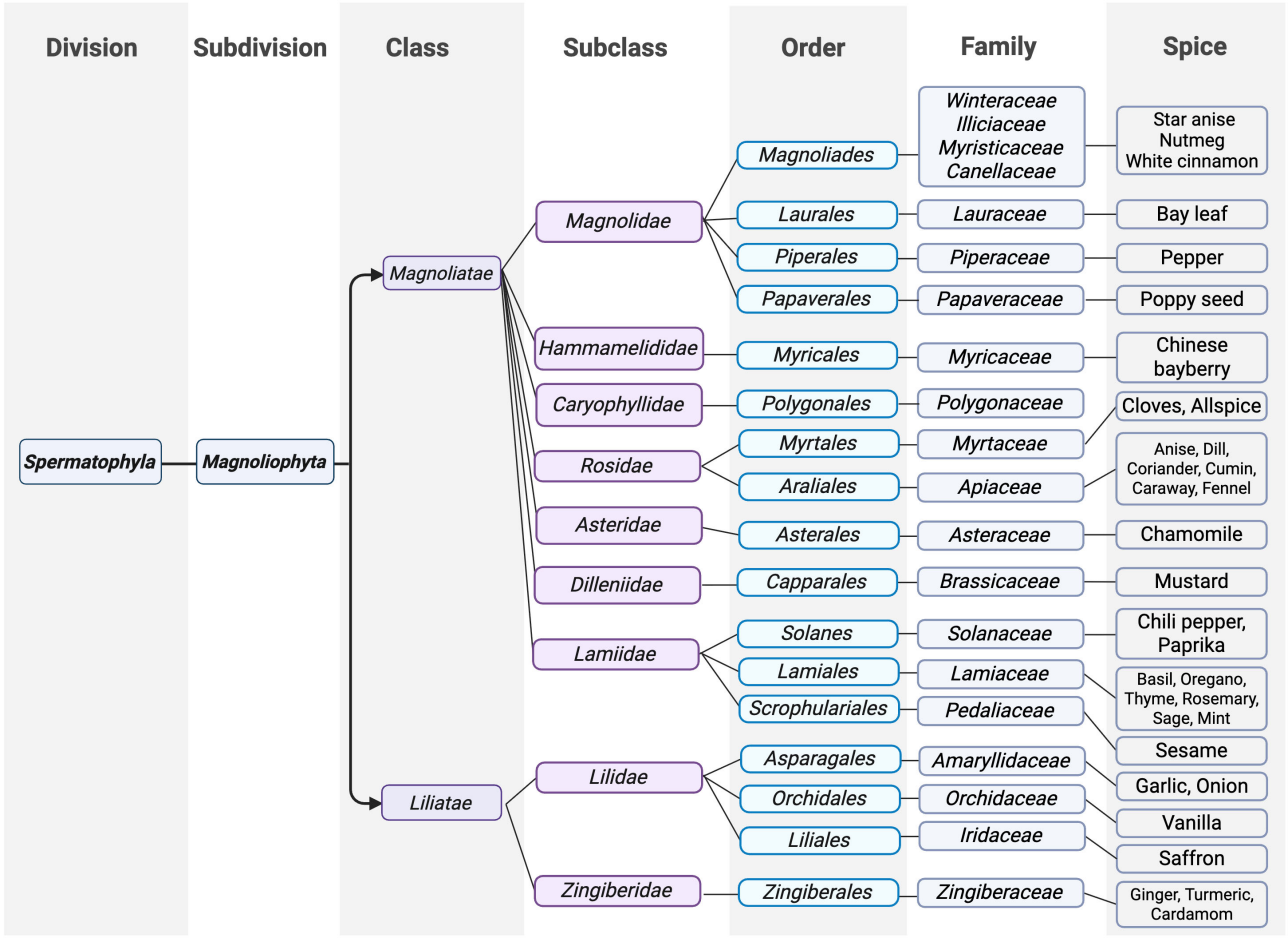
Botanical relation of the main spice families. Adapted from Schöll et al. (2). Created with BioRender.com.

The term “spice” refers to any plant-derived product used during cooking for food seasoning to add an aroma (1). Due to the global proliferation of ethnic cuisines, spices have become popular in both individual diets and food industries. Pepper, paprika, garlic, cumin, anise, oregano, and other spices are widely used in cuisine to enhance flavor profiles or serve as food preservatives. Spices are classified based on botanical analogies and taxonomic families (**Figure 1**) (2).

Similarly, condiments represent ready-to-use sauces, pastes, or mixtures (e.g., ketchup, mustard, soy sauce) added during or after cooking, and some of them also contain spices, rendering ingredient identification sometimes difficult (3).

Allergic signs and symptoms due to spice can be the result of Immunoglobulin E (IgE)- or non-IgE-mediated mechanisms (4). Moreover, severe irritative reactions (mediated by capsaicin) affecting the skin or respiratory tract are difficult to distinguish from immune-mediated reactions (5, 6). Some substances in spices like hot pepper (*Piperaceae*) and paprika, cayenne or chili (*Capsicum annuum*) could also increase the permeability of epithelia determining sensitization to proteins from other foods, below a molecular mass of 70 kDa (7). Regarding IgE-mediated spice allergy, no data are available about safe threshold doses, and very low amounts of proteins have been described to cause anaphylaxis (8).

Epidemiological data suggest that spice allergies are relatively rare, accounting for only 2–4% of all food allergies in adults. Adult women appear to be at higher risk, likely due to the use of cosmetics and fragrances. Current literature lacks studies on the prevalence of spice allergies in children. However, consumers constantly and unintentionally ingest undeclared natural flavors or spices, with the risk of severe reactions. Spice labeling is often inaccurate owing to varying national food regulations across countries. Furthermore, spices are frequently secret ingredients in traditional recipes and may cause reactions both upon ingestion and inhalation. In the European Union, Regulation No. 1169/2011 on the provision of food information to consumers has been effective since December 2014. According to Annex V, spices and herbs that constitute less than 2% of a finished product are declared generically as “spices” or “herbs” or “mixtures” thereof. Therefore, unlike mustard, celery, and lupin, which require mandatory declaration, as specified in Annex II, spices and herbs are often not specified in the final food product labeling (9).

In children, anaphylaxis to spices is documented as isolated case reports (**Table 1**). The European anaphylaxis registry reviewed 1970 cases of anaphylaxis in children (0–17 years) and 4% were linked to spices (curry, poppy, pepper, mustard seed, sunflower seed, and pumpkin seed) (29). In the updated registry, published in 2023, spices are responsible for 32 of 4468 food-induced anaphylaxis cases (0.7%) (30). In a retrospective analysis, Jiang et al. reported spices as the cause of anaphylaxis in 25 of 1501 cases in both children and adults (1.7%) (31). Several additional anaphylactic and systemic reactions to spices in children have been reported worldwide in retrospective studies, based upon clinical data records (32).

**Table 1:** Published case reports of spice anaphylaxis in adults **(A)** and in children **(B)**.

Spice	Species	Family	Patient details	Reference
A.Adult cases				
Coriander (cilantro)	*Coriandrum sativum*	*Apiaceae*	25 year-old male	(10)
Cumin seeds (black C., green C., white C.)	*Bunium persicum*, *Cuminum cyminum*	*Apiaceae*	68 year-old female	(11)
Dill	*Anethum graveolens*	*Apiaceae*	40 year-old female	(12)
Garlic	*Allium sativum*	*Alliaceae*	23 year-old female	(13)
Mustard (black m., brown m., Indian m., oriental m., white m.)	*Brassica nigra* *Brassica juncea* *Sinapis alba*	*Brassicaceae*	38 year-old female 47 year-old female Lack of data	(14) (15) (16)
Oregano (wild marjoram, oregan)	*Origanum vulgare*	*Lamiaceae*	45 year-old male	(17)
Poppy seed (opium p., garde p.)	*Papaver somniferum*	*Papaveraceae*	Lack of data Lack of data	(18) (19)
Thyme (garden thyme)	*Thymus vulgaris*	*Lamiaceae*	45 year-old male	(17)
Curry powder	Mixture of different spices (turmeric, cumin, cloves, ginger, cinnamon, coriander, cardamom, cayenne pepper and paprika)	*-*	26 year-old anaphylaxis to cardamom and fenugreek contained in curry powder	(20)
Paprika	*Capsicum annuum*	*Solanaceae*	Lack of data	(21)
B.Pediatric cases				
Coriander (cilantro)	*Coriandrum sativum*	*Apiaceae*	14 year-old female	(22)
Mustard (black m., brown m., Indian m., oriental m., white m.)	*Brassica nigra* *Brassica juncea* *Sinapis alba*	*Brassicaceae*	15 year-old female	(15)
Poppy seed (opium p., garde p.)	*Papaver somniferum*	*Papaveraceae*	16 year-old female	(23)
Curry powder	Mixture of different spices		15 year-old male	(24)
Cayenne and black pepper	*Capsicum annuum*	*Solanaceae*	17 month-old male	(25)
Fenugreek	*Trigonella foenum-grecum Linn.*	*Fabaceae*	14 years-old male 14 years-old male	(26) (27)
Sumac	*Rhus coriaria*	*Anacardiacae*	14 year-old female	(27)
American ginseng	*Panax quinquefolius*	*Araliaceae*	6 year-old female	(28)

In this article, we report the first case of cumin anaphylaxis caused by inhalation in a patient under 18 years of age and performed a narrative review of pediatric spice allergy cases published in the literature.

A literature search was performed using PubMed, MEDLINE, and EMBASE databases to identify articles concerning spice allergy and anaphylaxis in the pediatric population. Articles published between January 1989 and March 2024 were selected. The search strategy included the terms “spices” and “herbs” combined with “food allergy,” “spice allergy,” “IgE-mediated allergy,” and “anaphylaxis,” along with pediatric-related keywords (“child,” “pediatric,” and “adolescent”). Case reports, case series, retrospective studies, and prevalence studies were included in this analysis. Articles describing allergic reactions following spice ingestion or inhalation were considered.

We also described a case report of anaphylaxis upon the inhalation of cumin spices in a pediatric patient. Information was retrieved from the patients’ electronic medical records. Informed consent was obtained from the patient’s parents for the publication of this case report.

## 2 Case description

A 17-year-old girl of Moroccan origin living in Tuscany was admitted to the Meyer Children’s Hospital IRCCS Allergy Unit for asthma, allergic rhinitis, and IgE-mediated food allergy.

Previous skin prick tests (SPT) were positive for kiwi, house dust mites, dog and cat dander, grass pollen, eggs, cod, strawberries, walnuts, and latex. Nonetheless, she tolerated all ingested foods except kiwis and was prescribed a kiwi-free diet.

She regularly used inhaled corticosteroid-formoterol therapy and reported poor control of respiratory signs and symptoms, with at least one acute asthma attack per month. The patient’s family history was negative for atopy.

The girl had never touched or ingested cumin in the past; she had consumed and tolerated all other spices, including turmeric, saffron, cardamom, and coriander. Since the age of three, the girl had experienced multiple reactions characterized by itching, rhinitis, and sneezing associated with face flushing after cumin inhalation; all reactions occurred while meals were prepared by her mother.

At the age of 17, the girl was in the kitchen while her mother was cooking eggs with cumin, and she immediately showed shortness of breath associated with rhinitis, generalized itching, flushing, and eye and lip angioedema. She did not report any physical exercise, medication, or food consumption before cumin exposure. The girl was administered a single oral dose of levocetirizine (10 mg) and betamethasone (4 mg) at home immediately after the onset of clinical manifestations.

She then presented to the hospital emergency department (ED), where she appeared in good clinical condition but with mild dyspnea, generalized itching, lip edema, and bilateral wheezing with an oxygen saturation of 93%. Other vital signs were normal.

The reaction was treated in the ED with a single dose of intramuscular epinephrine (0.01 mg/kg/dose, maximum 0.5 mg), intravenous chlorphenamine (0.25 mg/kg/dose), hydrocortisone (10 mg/kg/dose), and saline solution infusion at the hospital, with complete signs and symptom remission within 3–4 hours. Blood tests showed normal blood counts, biochemical profiles, and C-reactive protein levels; tryptase was not tested, probably due to the clear anaphylactic symptoms in the ED.

After 1 month, she was re-evaluated at the Allergy Unit outpatient clinic and a prick-by-prick (PbP) test was performed using cumin powder obtained from the trituration of dried seeds; the patient showed a strong positive response (wheal = 9 mm). The positive and negative controls for the test were a histamine (10 mg/ml; Lofarma, Milan, Italy) and normal saline SPT, respectively.

The patient was advised to avoid ingesting cumin and kiwi and to always carry an epinephrine autoinjector after training for proper use.

Given the risk of anaphylaxis, an oral challenge with cumin was not performed.

**Figure 2:**
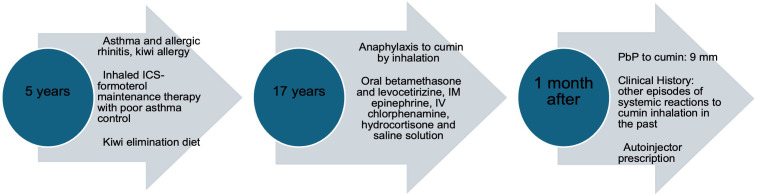
Summary of the clinical history of the patient and her diagnostic-therapeutic management over time. ICS, inhaled corticosteroid; IM, intramuscular; IV, intravenous; PbP, prick-by-prick.

The patient’s clinical history is summarized in **Figure 2**.

## 3 Discussion

In the literature, a wide spectrum of reactions to spices is described (33), mainly caused by food ingestion, predominantly documented in adults and sometimes associated with occupational exposure. Indeed, spice allergy is largely considered an occupational disease affecting the adult population (4), several case reports of anaphylaxis are documented in adults (10–14, 16–21, 34). In industrial settings, food workers may develop contact dermatitis or occupational asthma following inhalation of dust with garlic, onion, and chili pepper (35).

However, data on anaphylactic episodes following spice inhalation are limited. Chiu et al. reported an anaphylactic episode in a 40-year-old woman with a history of seasonal and perennial allergic rhinitis due to inhalation of food cooked with dill. Interestingly, in the past she complained of systemic symptoms after ingestion of this spice, and after repeated exposures, even by inhalation (12).

Although several foods can elicit anaphylaxis due to inhalation (36, 37), only one pediatric case report of anaphylaxis due to spice inhalation has been described: a 6-year-old girl with urticaria and respiratory symptoms after inhalation of powdered American ginseng (28). Our article reports the first case of anaphylaxis caused by cumin inhalation in a patient aged less than 18 years.

Among spices, cumin (*Cuminum cyminum L.*) belongs to the *Apiaceae (Umbelliferae)* family and its yellowish-brown seeds are valued for their aroma and warm taste in cuisine as well as for pharmaceutical applications (38). Being the second most used spice in the world, cumin rarely triggers allergic reactions, although isolated cases of anaphylaxis are reported in adults (11).

Generally, the most allergenic spices in children belong to the *Apiaceae* family, which includes also coriander, fennel, celery, chervil and dill, and the *Liliaceae* family, including garlic, onion, chives, shallot and saffron (5).

As reported by Cercle d’Investigations Cliniques et Biologiques en Allergologie Alimentaire, a French society for food allergy research, real sensitization to spices in children suspected of food allergy could be more common than we thought. In a study that included 589 cases of food allergy, 26/81 (32%) of children showed a positive prick test for *Apiaceae*. Notably, of the children tested for cumin, 20% were prick test-positive, while sensitization to Liliaceae (e.g. garlic, onion, and chive) was only 4.6%. Despite the high sensitization rate in children, no case of pediatric allergy to spice has been confirmed in this case series (39).

Severe allergic reactions to spices have been described in the pediatric population in several case reports, as well as mild-to-moderate reactions (**Table 2**).

**Table 2:** Published case reports of pediatric IgE-mediated allergies to spice and herbs.

Author	Spice type	Exposure	Age at onset & Sex	Clinical manifestations	Diagnostic tests	Other allergies	Reference
Gimenez L, et al. WMJ 2011	Cayenne and black pepper	Meat prepared with spice marinade By ingestion	17 month-old male	Severe anaphylaxis (urticaria, conjunctivitis, facial swelling, and severe cough)	•SPT with extracts of onion, garlic, paprika, thyme, black pepper, cayenne pepper, tomato, and crude extracts of mesquite, Southwest, Montreal and chipotle marinade (cayenne pepper 2x9 mm wheal; black pepper 3x14 mm wheal) •s-IgE to cayenne pepper: 0.11 kU/L	Egg allergy	(25)
Che CT, et al. J Allergy Clin Immunol Pract. 2017	Fenugreek	Curry By ingestion	14 year-old male	Anaphylaxis (Pruritus and tingling lips, chest heaviness, urticaria, and wheezing)	•SPT to 11 common components of curry (fenugreek: 13 mm wheal)	Peanut, lentil, chickpea and pea allergy	(27)
Che CT, et al. J Allergy Clin Immunol Pract. 2017	Sumac	Lebanese Fattoush salad (vegetables with za’atar: a mixture of sumac, sesame, dried thyme, and salt) By ingestion	14 year-old female	Two episodes of anaphylaxis (abdominal discomfort and hives with pruritus, cough, and eyelid angioedema)	•SPT to the common components of Fattoush salad (sumac: 15 mm wheal)	Cashew, pistachio peanut, and soy allergy	(27)
Joseph NI, et al. Allergy Rhinol. 2018	Fenugreek	Spread consisting of fenugreek, lemon, garlic, and cilantro By ingestion	14 year-old male	Anaphylaxis (hives, chest tightness, abdominal pain, and emesis)	•PbP with a solution of ground fenugreek: 15 mm wheal with pseudopods •s-IgE to fenugreek: 38.10 kU/L (reference <0.35)	Fava bean and lentil allergy	(26)
Ju Suk L, et al. J Allerg Clin Immunol. 2004	Curry	Curry powder with rice	15 year-old male	Immediate palatal itching, generalized urticaria, headache and dyspnea.	•OFC (positive at 20g of curry powder with water)	SPT positive for soybean, asparagus, weed pollens, flower pollens and HDM	(24)
Subiza J, et al. J Allergy Clin Immunol. 1989	Chamomile	Ingestion of a chamomile-tea infusion	8 year-old male	Anaphylaxis	•SPT to chamomile-tea extract •Specific antichamomile-tea extract and anti-*Matricaria chamomilla*-pollen extract IgE antibodies by ELISA technique	Bronchial asthma caused by a variety of pollens (grass, olive, mugwort)	(40)
González-de-Olano D, et al. J Investig Allergol Clin Immunol. 2018	Garlic	Accidental ingestion of homemade garlic sauce (first contact)	9 month-old breast-fed female	Generalized erythema and coughing	•PbP with garlic extract (10 mm wheal) •s-IgE to garlic: 3.15 kU/L •Immunoblotting: identification of lectin as main allergen		(41)
Fiocchi A, et al. Ann Allergy Asthma Immunol. 2014	Saffron	Saffron risotto By inhalation	12 year-old male	Sneezing immediately after smelling and eating a small amount of risotto	•SPT with rice: negative •PbP with saffron powder: 5 mm wheal	Allergic rhinitis to grass pollen, cat dander, and HDM Oral allergy syndrome with apple, watermelon and fennel	(42)
Yazıcı S, et al. Iran J Allergy Asthma Immunol. 2018	*Salvia officinalis, Mentha piperita* and *Origanum onites* L.	Chicken meat with cheddar cheese Sage tea By ingestion	13 year-old male	Angioedema	•PbP positive only for mint •OFC for sage, oregano and mint: positive		(43)
Eseverri JL, et al. Allergologia et Immunopathologia 1999	Paprika, cumin, anise, mustard	Not well defined	3 children By ingestion	Urticaria	•Not defined		(44)
Morisset M, et al. Allergy 2003	Mustard	Routine dose of mustard in DB- or SB-PCFC By ingestion	23% of 30 subjects aged 3–20 years (6 children aged 3-15 years) were allergic to a routine dose of mustard (1349 mg of seasoning)	Asthma, abdominal pain, atopic dermatitis, angioedema, and digestive symptoms	•SPT with ground *B. nigra* seeds: 6.9 mm wheal; with *B. juncea* flour: 7.8 mm wheal; with mustard seasoning: 9.7 mm wheal •s-IgE to mustard •DBPCFC		(45)
Mailhol C. et al. Eur J Dermatol. 2014	Mustard, Sesame	Not well defined	386 AD patients (age 0–18 years), 69 children with a final diagnosis of FA: mustard as culprit in 1% of FA cases	AD exacerbation	•4% of the cohort with positive SPT to mustard •OFC in positive SPT patients	26 patients with FA to multiple foods (not further described)	(46)
Poikonen S, et al. Acta Paediatr. 2009	Turnip rape Mustard	Labial challenge ➔ if negative OFC with crushed turnip rape seeds in children with AD and positive SPT to turnip rape OFC with mustard By ingestion	14 Finnish and 14 French AD patients with positive turnip rape SPT Turnip rape challenge: positive in 14/14 (100%) Finnish and 5/14 (36%) French children Mustard OFC: positive in 5/14 (36%) Finnish and 5/14 (36%) French children	Turnip rape challenge:- Finnish 12/14 positive labial, 2/14 facial urticaria- French 5/14 positive labialMustard OFC: - Finnish 3/14 facial urticaria, 2/14 abdominal pain and vomiting- French 2/14 urticaria, 1/14 rhinitis and asthma, 2/14 abdominal pain and vomiting	•SPT: Turnip rape and mustard •Specific IgE and enzyme-linked immunosorbent assay (ELISA) •Labial challenge •OFC	Egg as major associate allergen in both groups Birch in Finnish children	(47)
Rancé F, et al. Allergy 2000	Mustard	Mustard powder masked in stewed apple (progressive doses: 1, 5, 10, 20, 50, 100, 250, and 500 mg)	36 children with positive mustard SPT (10 months-15 years old)	Open or SBPCFC: 16/36 positive (14 urticaria, 3 rhinoconjunctivitis, 1 angioedema, 1 OAS, 1 eczema)	•SPT (mean 8.8 mm in allergic patients) •Open or SBPCFC •s-IgE (mean 23.8 kU/L in allergic patients)	Egg and peanut as major associate allergen in both groups Also kiwi fruit, avocado, soy, beef, wheat in the allergic group	(48)
Rancé F, et al. Arch Pediatr. 2002	Mustard	Not well defined	6,9% of 250 positive DBPCFC In asthmatic children with FA	AD, urticaria and angioedema	•SPT •DBPCFC		(49)
Meincke R, et al. Pediatr Allergy Immunol. 2017	Mixed herbal products	Mixed herbal products (51.4%), *Hedera helix* (15.0%), *Echinacea purpurea* (5.6%)	79 cases with 107 allergy-like reactions Routes of administration: oral (75.9%), topical (8. 9%), and rectal (3.8%) Age <18 years (mean 8.3 y)	59.8% urticaria or rash or erythematous rash Allergic reaction (8.4%)	•WHO global individual case safety report (ICSR) database VigiBase^®^ in children		(50)
Kanny G, et al. Allerg Immunol. 1994	Vanilla Balsam of Perù	Natural vanilla, artificial vanillin and Balsam of Perù By ingestion	11 children with severe AD (<5 years)	9/11 eczematous reactions, 1/11 Quincke’s oedema 2/11 negative OFC	•DBOFC with balsam of Peru (225 mg), natural vanilla (50 mg), artificial vanillin (12.5 mg)		(51)
Admani S, et al. Pediatr Dermatol. 2017	Cinnamon	Homemade cinnamon sugar scrub (via skin contact) and spice added to desserts (via ingestion)	16 year-old female with AD	Severe AD exacerbation	•Epicutaneous patch test positive 2+ to cinnamon extract		(52)
Barzegar S, et al. World Allergy Organ J. 2010	Spices (not specified)	Record of all the anaphylaxis cases in an Iranian Allergy unit	2 children Not defined sex and age	Anaphylaxis	•Retrospective study of anaphylactic episodes		(32)
Jiang N, et al. Allergy Asthma Immunol Res. 2016	Spices (not specified)	Retrospective analysis of anaphylaxis cases in a Chinese tertiary allergy unit	7 cases (3/7 aged 4–9 years, 4/7 10-17 years)	Anaphylaxis	•Retrospective study of anaphylactic episodes		(31)
Grabenhenrich LB, et al. J Allergy Clin Immunol. 2016	“Other spices” including curry, poppy, pepper, mustard, sunflower seed, and pumpkin seed	Retrospective analysis of European Anaphylaxis Registry (from 10 European tertiary hospitals)	12 cases of known allergy (5/12 <6 years, 3/12 6–12 years, 4/12 13–17 years)	Anaphylaxis	•Retrospective study of anaphylactic episodes		(29)
Bock A S, et al. J Allergy Clin Immunol. 1993	Coriander	Chicken in teriyaki marinade (ingredients: pepper extract, capsicum extract, ginger, coriander, and a cooking wine) By ingestion	14 year-old female	Anaphylaxis	•Skin tests with ginger, pepper, and coriander with fresh material obtained by the patient from a grocery store: negative except coriander (7 mm wheal)	Seasonal rhinitis	(22)
Keskin O, et al. Allergy and Asthma Proceedings 2006	Poppy seed	Food with poppy seed By inhalation	16 year-old male	Anaphylaxis	•SPT •s-IgE (3.36 kU/L)	Allergy to hazelnut and peanuts, asthma	(23)
Erdle SC, et al. Allergy Asthma e Clinical Immunol. 2018	American ginseng	American ginseng By inhalation	6 year-old female 3 year-old male	Anaphylaxis (girl) Conjunctivitis (boy)	•SPT with American ginseng powder dissolved in water •Basophil activation test •OFC (boy)	Food allergies (girl) Atopic dermatitis (both) Asthma (boy)	(28)

AD, atopic dermatitis; DBOFC, double-blind oral food challenge; DBPCFC, double-blind placebo-controlled food challenge; SBPCFC, single-blind placebo-controlled food challenge; FA, food allergy; HDM: house dust mite; PbP, prick-by-prick; OFC, Oral food challenge; s-IgE, specific IgE, SPT: skin prick test.

For instance, an anaphylactic reaction after eating venison prepared with a marinade containing various spices, unveiled allergy to black and cayenne pepper in a 17-month-old child (25). Moreover, two cases of anaphylaxis after the ingestion of curry and pastes containing fenugreek have been described in children. Joseph et al. reported a case of fenugreek anaphylaxis in a 14-year-old boy who developed hives, chest tightness, abdominal pain, and vomiting after eating a spread made of fenugreek, lemon, garlic, and cilantro (26). Ju Suk et al. reported a curry allergy in a 15-year-old boy who presented with immediate palatal itching, generalized urticaria, headache, and dyspnea after eating aromatized rice with curry, confirmed by oral food challenge (24). Che et al. also reported the case of a 14-year-old male who developed anaphylaxis (pruritus, chest heaviness, urticaria and wheezing) after eating curry made with fenugreek (27). The same article reports anaphylaxis to sumac in a 14-year-old female shortly after eating a salad containing za’atar, a mixture of sumac, sesame, dried thyme and salt, typical of Lebanese cuisine (27). Other authors reported a case of an 8-year-old boy who experienced anaphylaxis after consuming a chamomile-tea infusion (40). Among the less severe reactions, a breastfed 9-month-old baby who accidentally ingested homemade garlic sauce for the first time started coughing and exhibited generalized erythema, most likely because the mother’s milk acted as the primary sensitizer. Positive PbP tests with the same sauce were demonstrated in the infant (41).

However, spice allergy may be difficult to hypothesize and investigate, even more so in children (53).

First, the diagnostic workup is often challenging because of a history of ingestion of a mixture, raising the possibility of multiple putative allergens. Indeed, some composite products (food and beverages) can contain spices and herbs that are not included in the ingredient list owing to their small quantity. For example, coriander used as a natural flavoring in beer has been linked to anaphylaxis in a case report, and it was not declared in label; this may represent a problem in adolescent-adult age (54). On the contrary, herbs and spices are often used in phytotherapy in infant products, and mustard and lupin could be found in pre-packaged foods for toddlers until the Legislation was changed (55). Moreover, due to allergen similarity, hidden allergens could be masked in spice: undeclared peanut and almond in a taco spice were subsequently found in cumin, as contaminants (56).

Our patient recognized cumin as causative of the reaction by herself; despite this, we underline the importance of a detailed medical history that evaluates all ingredients and ethnic habits, highlighting the role of the prick test with spice powder, as in our case, to avoid the improper diagnosis of idiopathic anaphylaxis and the risk of future reactions. Furthermore, the identification of spice allergies could be very difficult because of the widely varying laws governing food labeling; spices may not be described in detail in both traditional recipes and ready-made meals.

Second, the diagnostic process for spice allergies can be difficult. Muhlemann et al. demonstrated that prick tests with native spices and spice extracts produced comparable results, whereas s-IgE testing was negative in more than half of the cases (57). In other studies, the sensitivity of the prick test using extracts changed considerably; Niinimaki et al. reported a higher rate of positivity when skin tests were performed with extracts containing a 5% concentration of spice (weight to volume) (58, 59). Better standardization of the method is necessary to minimize false-positive results using native species for the PbP test, which is considered a potential irritant capacity for some of them. Some studies emphasized the role of native prick tests with herbs and spices in the diagnostic workup of pediatric patients with pollinosis, encouraging larger clinical studies to investigate the clinical impact of herb sensitization in children (60).

In conclusion, studies have focused on the sensitivity and specificity of skin tests (both with native spices and extracts) with variable results, and blood tests for spices are missing. Most studies are on a small sample, mostly in adults without a documented history of spice allergy. Moreover, experts consider that there are currently limited data regarding the reliability of skin tests and s-IgE; therefore, when the suspicion of spice allergy is strong, despite negative test results, a double-blind placebo-controlled food challenge with powdered spices entrapped in capsules should be performed (45). Notably, although several studies addressing skin tests and *in vitro* assays date back more than two decades, these limitations persist. Modern, standardized diagnostic tools for spice allergies in children may be available, but they are not yet validated or widely used. One example is the use of multiplex tests, which represent a promising diagnostic frontier, offering simultaneous testing for multiple spices (e.g., anise, cumin seeds, oregano, paprika, and parsley). Unfortunately, multiplex tests, as well as the basophil activation test, were not available for our case report at the time of writing.

Third, sensitization to spices could also happen after primary sensitization by inhalation of pollen and following a cross-reaction, especially with birch or wormwood (such as in celery-mugwort-spice syndrome) (61–63). Wagner et al. recently demonstrated a stronger correlation in children than adults, even a two-to-four-fold higher, between prick test positivity for *Lamiaceae* or *Apiaceae* and pollinosis (birch, mugwort or grass pollen), although spice allergy symptoms in the sample have not been studied (60). In his study, pediatric patients with pollinosis responded 16–18 times more often to skin tests with herbs of *Apiaceae* family and 28 times more often to lemon balm (*Lamiaceae* family) than patients without pollinosis.

In these patients, inhalation of allergenic proteins could be the main mechanism of sensitization, since Bet v 1 homologues and profilins in *Apiaceae* and *Solanaceae* families are labile to high temperature and sensitive to gastric digestion, and determine cross-reactions between spices and vegetables within the same family, such as celery, carrot, and many popular spices, such as cumin, anise, and fennel.

Indeed, cross-reactions are more frequent among spices from the same phylogenetic tree (2, 4). Most of the reactions to anise, dill, coriander, and cumin are caused by allergenic determinants which belong to the family of Bet v 1 and profilin homologues like in cumin (Cum c 1 and Cum c 2), fennel (Foe v 1 and Foe v 2), coriander (Cor s 1 and Cor s 2) and anise (Pim a 1 and Pim a 2) (64), but also to other allergens like the defensin like protein 1 in celery (Api g 7) (61). Moreover, in 2020, Slowianek et al. identified new allergens in anise and caraway, respectively glyceraldehydes-3-phosphate dehydrogenase and elongation factor α, together with other cumin IgE-binding proteins with variable molecular mass (of 54, 42, 38, 31, and 20 kDa) (65).

Concerning the extent and type of processing, spice allergens in food may be degraded, but enhancement of the IgE-binding capability is sometimes possible (2). In hot spice paprika, high temperature destroys Bet v 1 homologues, but an osmotin named Protein P23 was demonstrated to be heat-resistant (66). Jensen-Jarolim et al. proved that dried *Apiaceae* spices still contain intact Bet v 1 and profilin homologues (64). In *Apiaceae* and *Solanaceae* families, cross-reactive carbohydrate determinants are also expressed, which are more resistant to food processing, including grinding and roasting (2, 64).

Moreover, due to the presence of seed-storage proteins (such as mustard, sesame, peanut, Brazil nut, and walnut), other spice types may also cause cross-reactions with different foods. The 7S-vicilin and 11S-legumin allergens revealed considerable homologies to peanut Ara h 1 and Ara h 3, respectively, explaining clinical cross-reactivity between peanut and fenugreek (67). Being fenugreek part of the *Leguminosae* family, allergic patients can be cross reactive to other legumes, like fava beans and lentils (68).

The current perception of plant-based foods and food-flavoring agents as innocuous and the increasing use of ethnic and creative cuisines in the pediatric population may affect the future prevalence of this rare entity (69). Therefore, spice variability warrants further molecular research to identify all allergic determinants, assess the reliability of the prick test considering the different irritant capacities of the spice, and highlight the need for a registry of pediatric spice allergy cases to better understand its potential clinical impact. Clinical history should always guide subsequent investigations, and spice allergies in children may be difficult to suspect unless careful anamnestic investigation is performed. Multiplex tests may further aid in identifying such cross-reactivity and profiling individual risk to the patient. Finally, follow-up clinical studies in pediatric populations are needed to understand the long-term prognosis of spice allergies.

## Data Availability

The original contributions presented in the study are included in the article. Further inquiries can be directed to the corresponding author.
